# Serological investigations of Peste des Petits Ruminants in cattle of Nepal

**DOI:** 10.1002/vms3.354

**Published:** 2020-09-14

**Authors:** Meera Prajapati, Swoyam Prakash Shrestha, Dipak Kathayat, Yongxi Dou, Yanmin Li, Zhidong Zhang

**Affiliations:** ^1^ State Key Laboratory of Veterinary Etiological Biology Lanzhou Veterinary Research Institute Chinese Academy of Agricultural Sciences Lanzhou China; ^2^ CAAS‐ILRI Joint Laboratory for Ruminant Disease Control Lanzhou Veterinary Research Institute Chinese Academy of Agriculture Sciences Lanzhou China; ^3^ National Animal Health Research Centre Nepal Agricultural Research Council Lalitpur Nepal; ^4^ Food Animal Health Research Program Department of Veterinary Preventive Medicine The Ohio State University Columbus OH USA

**Keywords:** cattle, Nepal, PPR, seroprevalence

## Abstract

Peste des Petits Ruminant (PPR) is an infectious viral disease of small ruminants caused by PPR virus. Although goat and sheep are the primary hosts of PPR, studies have continuously reported the prevalence of circulating antibodies in large ruminants, which could bring a potential challenge to effectively control and eradicate PPR. In Nepal, seroprevalence of PPRV antibodies in cattle have not been monitored yet. To address this, a total of 255 cattle sera were collected from Rupandehi, Banke, Bara and Chitwan districts of Nepal where outbreak of PPR in small ruminants was reported previously. The sera samples were tested by competitive ELISA and the result indicated the prevalence of 5.88% PPRV antibodies in cattle which indicates the exposure of cattle to PPR virus. To make the disease control program effective, intensive monitoring of both domestic and wild animals is very important.

## Graphical abstract

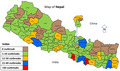

## 1 INTRODUCTION

Peste des Petits Ruminant (PPR) is an important transboundary viral disease of small ruminants caused by PPR virus (PPRV). The disease is manifested in sheep and goats causing fever, diarrhoea, conjunctivitis, rhinotracheitis, ulcerative stomatitis, gastroenteritis and pneumonia (Taylor, 1984). The morbidity and mortality rate may reach up to 100% and 90%, respectively (Pope et al., 2013). Other unusual hosts have also been reported to be infected by PPRV which may obstruct in control and eradication of PPR globally. PPRV was first reported in Ivory Coast (Cote d'Ivoire) of West Africa in 1942 (Gargadennec & Lalanne, 1942). But, as the disease is highly contagious and infectious, it has now spread worldwide including Central and East Africa, the Middle East, Turkey, China, India and Nepal reaching Europe doorstep with cases reported in Morocco (Baazizi et al., 2017), Turkey (Sevik & Sait, 2015) and Georgia (Donduashvili et al., 2018).

**FIGURE 1:**
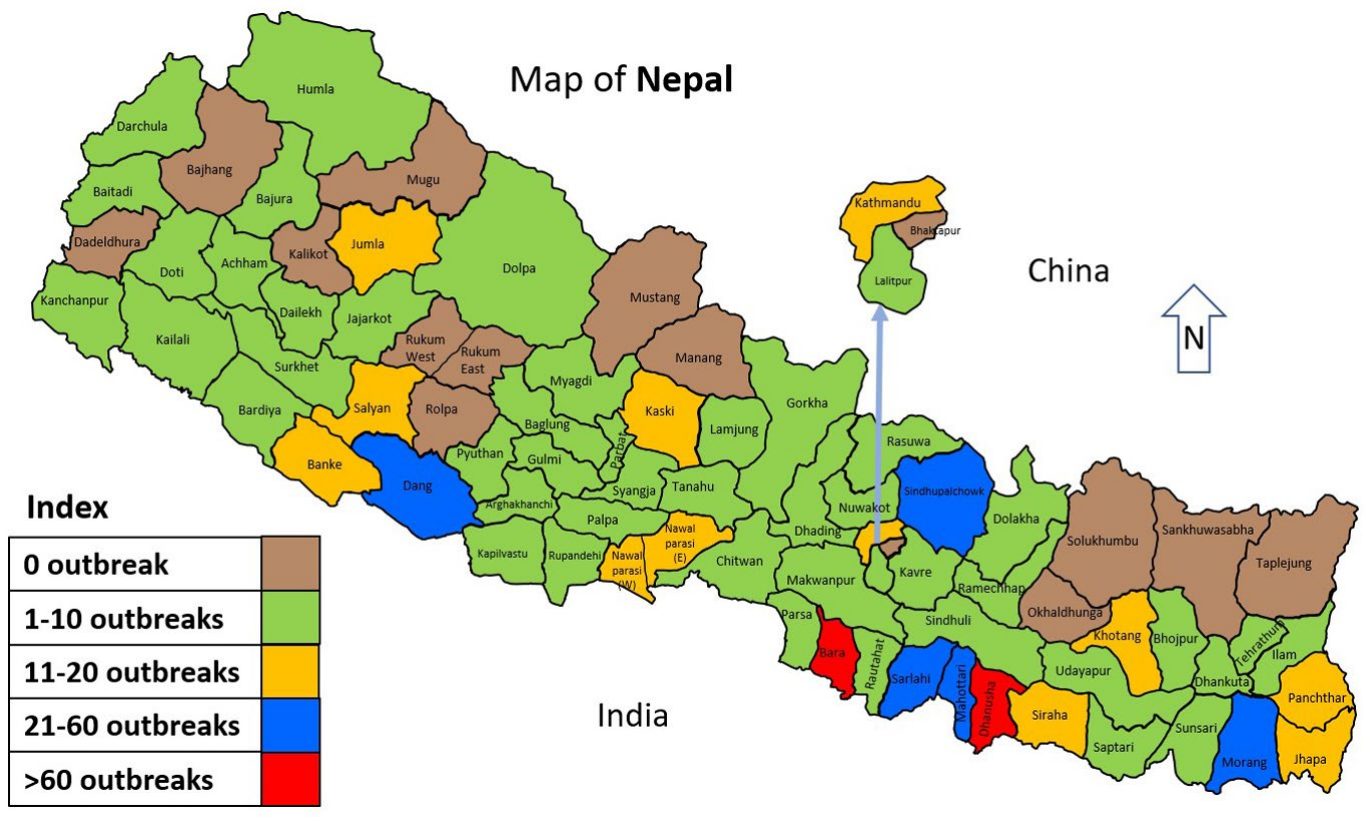
District‐wise distribution of PPR in Nepal Source: Situation analysis of Peste des Petits Ruminants (PPR) for past 10 (2008–2017) years in Nepal, Veterinary Epidemiology Section, Animal Disease Investigation and Control Division, Tripureshwor, Kathmandu

In Nepal, the disease was first reported in 1995 from Dhanusha, Mahottari, Bara, Sarlahi, Rautahat and Gorkha districts of Nepal (Banyard et al., 2010; Dhar et al., 2002). Since then, the disease is widespread in Nepal and every year outbreak of disease occurs. Moreover, there is a great possibility that many outbreaks of disease might go underreported due to the absence of PPR specific surveillance programs in Nepal and therefore the real incidence of PPR might be higher. Out of 77 districts, the disease has spread to 71 districts in Nepal (Veterinary Epidemiology Section, 2018). Outbreaks of PPR in sheep and goats from 2008 to 2017 have been detailed in Table 1. Likewise, district‐wise distribution of PPR outbreak is shown in Figure 1. According to the data from 2008 to 2017, out of 77 districts, outbreaks were reported from 60 districts, among which the higher number of outbreaks were reported from Dhanusha (214) and Bara (83) districts of Province 2, respectively.

**TABLE 1:** Temporal pattern of PPR outbreaks in sheep and goats during 2008–2017

Year	No. of outbreaks	No. of affected	No. of deaths	No. of affected animals per outbreak (average)	Average deaths per outbreak
2008	77	635	172	8.25	2.23
2009	143	3,589	953	25.10	6.66
2010	181	6,374	1,166	35.22	6.44
2011	138	5,968	893	43.25	6.47
2012	78	3,073	639	39.40	8.19
2013	3	128	25	42.67	8.33
2014	50	17,573	3,914	351.46	78.28
2015	71	21,266	7,405	299.52	104.30
2016	56	14,252	2,449	254.50	43.73
2017	37	770	346	20.81	9.35

For the prevention and control of disease, national PPR control program has been launched since 2001 to till date. However, illegal trade of animals through open border between India, poor biosecurity and quarantine measures in farms and livestock markets, and lack of animal identification and tracking system has caused problem in the control and eradication of disease. In addition, stringent epidemiological surveillance of the disease is extremely important. Surveillance should be done not only to the primary hosts but also to other unusual hosts. Besides goat and sheep, studies have continuously reported the prevalence of circulating antibodies in cattle, buffalo, camel and wild animals as well (Abraham et al., 2005; Anderson & McKay, 1994; Balamurugan et al., 2012, 2014; Lembo et al., 2013). The role of these animals in virus transmission and maintenance remains unclear. Lembo et al. (2013) and Sen et al. (2014) have reported the isolation of PPRV from subclinically infected cattle. The role of cattle in the epidemiology of the disease is vague and needs to be investigated in detailed. In Nepal, seroprevalence of PPRV antibodies in cattle have not been monitored yet. To address it, this study was conducted.

## 2 METHODS

**FIGURE 2:**
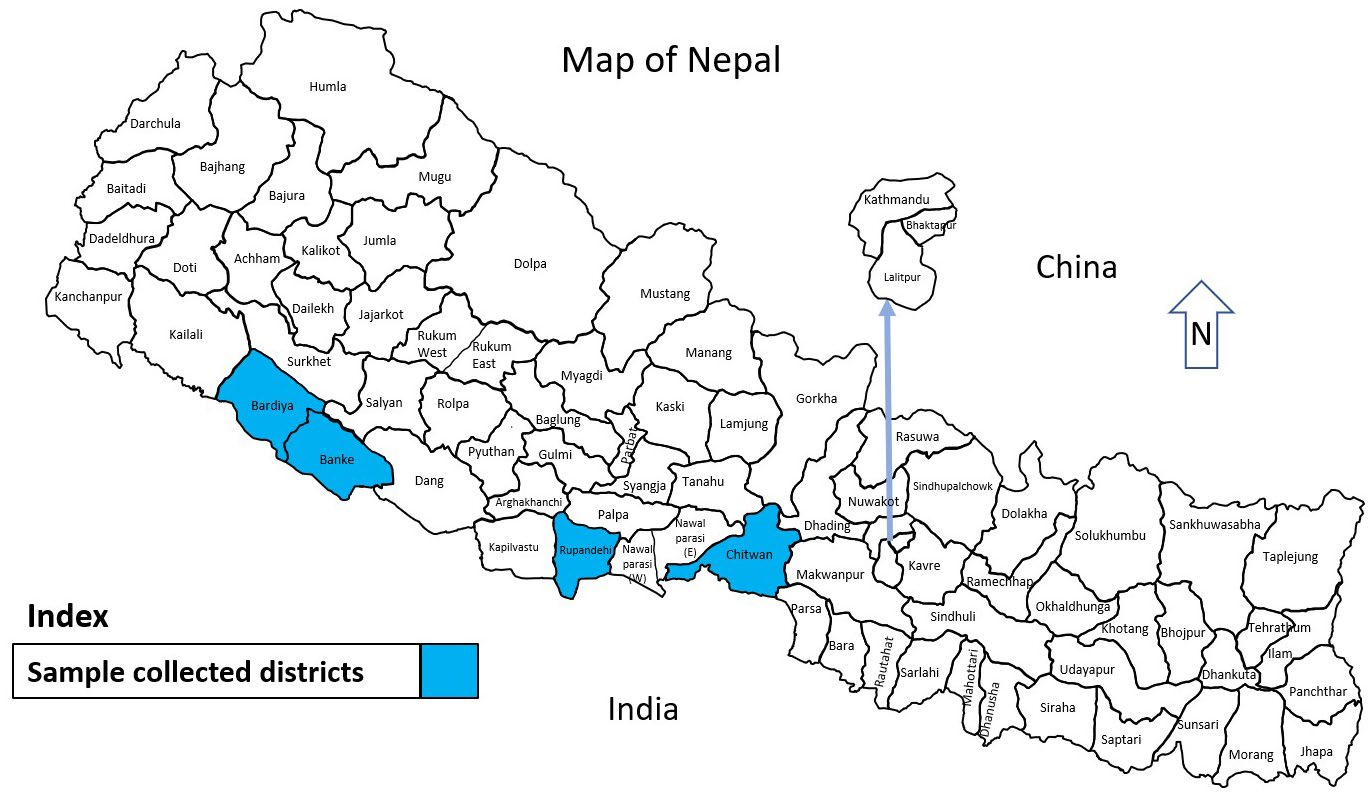
Map of Nepal with sample collected districts

Blood samples of cattle were collected from four districts of Nepal where outbreak of PPR was reported previously using 10‐ml sterile syringe. The syringe was kept undisturbed with the needle‐holding end positioned until the blood clots and the clear serum was separated. A total of 255 cattle sera were purposively collected from Rupandehi (92), Banke (45), Bardiya (47) and Chitwan (71) districts of Nepal (Figure 2) with the approval of the farmers and the concerned veterinary officer. The samples were then stored at −40°C for future test regardless of their age, sex and breed.

Then, a competitive enzyme‐linked immunosorbent assay (C‐ELISA) was performed for detection of PPRV antibodies in the collected sera samples using a competitive screening ELISA kit ID Screen® PPR Competition kit (IDVet Innovative Diagnostics) following the manufacturer's instructions. The prevalence rate was calculated as:

$$S/N\%=\left({\text{OD}}_{\text{sample}}/{\text{OD}}_{\text{NC}}\right)\times 100,$$

where OD_sample_ is the OD value of sample and OD_NC_ is OD value of negative control.

Samples presenting an S/N%: less than or equal to 50% are considered positive, greater than 50% and less than or equal to 60% are considered doubtful and greater than 60% are considered negative as per the protocol. The average diagnostic specificity and relative sensitivity of the assay is estimated to be 97.98% and 93.95%, respectively (Acharya, Poudel, & Acharya, 2018). The statistical analysis was carried out using Fisher's exact test. *p* value less than 0.5 was considered for statistical significance.

## 3 RESULTS

In Rupandehi, Chitwan, Banke and Bardiya, the seroprevalence rate was found to be 8.69%, 4.22%, 4.44% and 4.25%, respectively, as shown in Table 2. The overall seroprevalence of PPR in cattle was found to be 5.88% (Table 2). Statistical analysis using Fisher's exact test showed no any significant difference in seroprevalence of PPR antibodies according to location (*p*‐value > 0.05; Table 3).

**TABLE 2:** Seroprevalence of PPR in cattle according to location in Nepal

Location	Positive (%)	Negative (%)	Doubtful	Total	CI (positive)	CI (negative)
Rupandehi	8 (8.69)	78 (84.78)	6	92	(3.15–14.24)	(79.23–90.33)
Banke	2 (4.44)	41 (91.11)	2	45	(−1.43 to 10.32)	(85.23–97.00)
Bardiya	2 (4.25)	43 (91.49)	2	47	(−1.38 to 9.90)	(85.85–97.13)
Chitwan	3 (4.22)	65 (91.55)	3	71	(−0.35 to 8.80)	(86.97–96.12)
Total	15(5.88)	227 (89.01)	13	255		

Abbreviation: CI, 95% confidence interval.

**TABLE 3:** Statistical analysis of seroprevalence according to location using Fisher's exact test

Location	*p*‐value	OR
Rupandehi | Banke	0.4944	2.103
Rupandehi | Bardiya	0.4928	2.205
Rupandehi | Chitwan	0.3483	2.222
Banke | Bardiya	1.00	1.049
Banke | Chitwan	1.00	1.057
Bardiya | Chitwan	1.00	1.008

Note

*p*‐value < 0.05 is considered significant.

Abbreviation: OR: odds ratio.

## 4 DISCUSSION

Cattle of Nepal has never been vaccinated against PPR and rinderpest. Rinderpest has been eradicated from Nepal and worldwide, therefore the seroprevalences could have resulted from field infection with PPR virus. Even though the sample size is very small, detection of PPRV antibodies in cattle indicated that these animals were exposed to PPRV via contact with infected small ruminants which might lead to subclinical infection. Likewise, the prevalence rate of PPRV antibodies in various kinds of livestock at different geographical location with varied agro climatic condition should be investigated for knowing the status of disease under natural condition which might help in disease control strategies and implementation of vaccination program. This study has provided the baseline data on the prevalence of antibodies in cattle of different districts of Nepal. Seroprevalence studies of PPRV antibodies in cattle were reported in several countries. Balamurugan et al. (2012) showed the overall prevalence of 4.58% PPRV antibodies in 2,159 serum of cattle and buffaloes; and in 2014, the seroprevalence was found to be increased to 11.07% and 16.2% in 1,498 serum samples of cattle and buffaloes, respectively (Balamurugan et al., 2014). The higher seroprevalence of 41.86% in cattle and 67.42% in buffaloes was reported from Pakistan (Khan, Siddique, Sajjad Ur, Abubakar, & Ashraf, 2008). Similarly, a study of 1,000 sera samples of cattle showed the higher seroprevalence of 42% in Sudan when tested by competitive ELISA (Ali et al., 2019). Besides, cattle and buffaloes, PPRV antibodies has also been documented from camels, lions and other wild animals. The seroprevalence study of PPRV antibody in camels, cattle, goats and sheep showed the higher prevalence of 13% in sheep and lowest in camel (3%; Abraham et al., 2005). Likewise, seroprevalence of PPR in camels has also been reported from different countries. Woma et al. (2015) reported the prevalence of 3.36% of PPRV infection in camels in Nigeria. Studies have documented the presence of PPRV antibodies in cattle sera from Tanzania, Bangladesh, Pakistan, India, Ethiopia and Nigeria (Abraham et al., 2005; Abubakar et al., 2017; Balamurugan et al., 2012; El‐Yuguda, Baba, Abdul, & Egwu, 2013; Haque et al., 2004; Lembo et al., 2013; Rashid, Asim, & Hussain, 2008; Razzaque et al., 2004). However, even though, there is no any evidence of transmitting the disease to sheep and goats, the role of cattle in the epidemiology of the disease remains questionable. In this study cattle sera were collected from those districts where PPR outbreaks were reported previously and therefore cattle could have been infected from small ruminants. Even though, there are many reports of circulating PPRV antibodies in other unusual host in different countries, this is the first study to report the presence of PPRV antibodies in cattle in Nepal. Due to time and diagnostic kit limitations, only few samples were screened for PPRV in this study. To make the PPR control program effective, further screening should be done in cattle, buffalos, pigs and wild ruminant animals of Nepal. These animals should be included in the sero‐monitoring program of PPR to give a better indication of the national herd immunity and to assess in the ongoing eradication program.

## Ethics statement

The authors confirm that the ethical policies of the journal, as noted on the journal's author guidelines page, have been adhered to. No ethical approval was required as no animal was treated in this study.

## CONFLICT OF INTEREST

The authors declare no conflict of interest.

## AUTHOR CONTRIBUTION

**Meera Prajapati:** Conceptualization; Methodology; Writing‐original draft; Writing‐review & editing. **Swoyam Prakash Shrestha:** Resources; Supervision. **Dipak Kathayat:** Data curation. **Yongxi Dou:** Resources; Supervision. **Yanmin Li:** Supervision; Writing‐review & editing. **Zhidong Zhang:** Funding acquisition; Resources; Supervision; Writing‐review & editing.

### PEER REVIEW

The peer review history for this article is available at https://publons.com/publon/10.1002/vms3.354.

## Data Availability

Available upon request to the corresponding author.
